# The effects of age and gender (bull vs steer) on the feeding behavior of young beef cattle fed grass silage

**DOI:** 10.5713/ajas.18.0698

**Published:** 2019-01-04

**Authors:** Natalia Puzio, Cezary Purwin, Zenon Nogalski, Ireneusz Białobrzewski, Łukasz Tomczyk, Jacek P. Michalski

**Affiliations:** 1 Department of Animal Nutrition and Feed Science, University of Warmia and Mazury, Olsztyn 10-719, Poland; 2 Department of Cattle Breeding and Milk Evaluation, University of Warmia and Mazury, Olsztyn 10-719, Poland; 3 Department of Systems Engineering, University of Warmia and Mazury, Olsztyn 10-718, Poland; 4 Department of Safety Engineering, University of Warmia and Mazury, Olsztyn 10-719, Poland; 5 The Kielanowski Institute of Animal Physiology and Nutrition, Polish Academy of Sciences, Jabłonna 05-110, Poland

**Keywords:** Bulls, Cattle, Feeding Behavior, Grass Silage, Steers

## Abstract

**Objective:**

The objective of this study was to determine the effects of age and gender (bull vs steer) on feeding behavior parameters in young beef cattle fed grass silage.

**Methods:**

The study was conducted on 180 young beef cattle at 7 to 18 mo of age. The experimental materials comprised 90 bulls produced by commercial crossing of Polish Holstein-Friesian cows with Charolais, Limousin and Hereford bulls (30 animals of each breed) and 90 steers of the same genotypes. The animals had *ad libitum* access to grass silage; the concentrate was fed separately, in feed stations. They received 28 g dry matter of concentrate per kg of metabolic body weight per day. Bunk visit data and silage intake for all experimental animals were recorded individually using the Roughage Intake Control system (5 feed bunks per 15 animals).

**Results:**

Age and gender (bull vs steer) exerted significant effects on the feeding behavior of young beef cattle. The frequency of bunk visits and meal frequency decreased, whereas the feeding rate of silage, and the average duration and size of a single meal increased with age (p<0.01). Bunk attendance and meal frequency were higher (p<0.01) in steers than in bulls (49.1 vs 37.4 visits/d, and 8.63 vs 7.99 meals/d, respectively). Daily feeding time was longer in steers than in bulls (102.3 vs 100.3 min/d, respectively), but the feeding rate of silage was lower in steers, and their meals were smaller in size and shorter in duration (p<0.01). Daily silage dry matter intake was higher (p<0.01) in bulls than in steers (4.62 vs 4.47 kg/d, respectively).

**Conclusion:**

The results of this study indicate that age and gender (bull vs steer) exerted significant effects on the feeding behavior of young beef cattle.

## INTRODUCTION

The feeding behavior and temperament of beef cattle may influence feed intake and overall performance. Therefore, a better understanding of feeding behavior could contribute to improving feeding strategies [1] and feeding behavior parameters should be controlled as part of nutritional management [2]. According to Lancaster et al [3], feeding behavior traits such as feeding duration, feeding frequency and time spent at the feeding station are significantly correlated with residual feed intake (RFI) and should be included in RFI models. Nkrumah et al [4] demonstrated that feeding behavior patterns should be considered when defining cattle breeding goals. Changes in feeding behavior can also serve as indicators of the health status of animals [2,5].

The most important determinants of feeding behavior in cattle are body weight (BW), age, breed, gender (bull vs steer) and individual characteristics of animals [6–8]. The main environmental factors affecting feeding behavior include feeding system, and the type, quality and availability of feed [1,2,5].

Most studies investigating feeding behavior in beef cattle housed in free-stall barns have involved animals fed a total mixed ration [9,10]. The intake patterns of grass silage, which is the most common feed for beef cattle in Poland, have been sporadically studied in dairy cows [11] and in-calf cows [12], whereas feed intake and feeding behavior in beef cattle offered grass silage remains poorly researched [13]. In view of the above, the objective of this study was to determine the effects of age and gender (bull vs steer) on feeding behavior parameters in young beef cattle housed in a free-stall system and fed grass silage *ad libitum* and small amounts of concentrate offered separately.

## MATERIALS AND METHODS

The animal handling and sampling procedures used in this study had been approved by the Local Ethics Committee for Animal Experiments in Olsztyn, Poland (decision No. 121/2010).

### Animals and management

The observations were carried out over 12 mo (from 7 until 18 mo of age) on 180 beef cattle. The animals were produced by commercial crossing of Polish Holstein-Friesian cows with Charolais, Limousin and Hereford bulls. Sixty crossbred male calves were produced in each crossing variant. Calves were purchased at 2 or 3 wk of age from different locations. Homogenous groups of calves in terms of sire breed were created within 10 to 14 days. After purchase, half of the male calves (30 animals per crossing variant) were castrated without anesthesia using industrial rubber elastrator rings, in accordance with the generally accepted standards. During the milk feeding period, from 2 wk of age, the calves received concentrate and hay *ad libitum*. Chemical dehorning was carried out in all animals at 8 wk of age. When the milk feeding period was completed and the calves reached average BW of 125 kg (group’s mean value), they were fed grass haylage *ad libitum* and concentrate in the amount of 2 kg per animal per day. At 7 mo of age, the animals were placed in a free-stall barn and allocated to pens (15 animals per pen) in homogenous groups based on gender (bull vs steer), age/BW and sire breed. The pens consisted of a straw-bedded area (12×7 m) and a standing alley (8×3 m) with feed bunks. All pens were identical, located in the same barn and equipped with one water trough (ID80, JFC Group, Karpin, Poland), one concentrate feed station (Insentec, Marknesse, Netherlands) and five roughage intake control (RIC) feed bunks (Insentec, Netherlands). Each feed bunk was 0.8 m wide, 0.75 m high, and 0.74 m deep. The animals had permanent access to water and salt licks (Lisal M, KSK, Kłodawa, Poland). Behavioral data were collected until the animals reached the age of 18 mo. In order not to disturb the existing hierarchy and to prevent aggressive behavior, the arrangement of the groups was not changed throughout the fattening period.

The animals had *ad libitum* access to grass silage; the concentrate was offered separately in the concentrate feed station. Experimental silage was made from a mixture of first-cut grasses (*Lolium perenne*, *Phleum pratense*, *Festuca rubra*, *Poa pratensis*), which were wilted for 24 h under favorable weather conditions, harvested using a precision chop forage harvester (7050, John Deere, Moline, IL, USA) and ensiled in horizontal silos without additives. Silage was removed from the silo using a self-propelled feed cart with a tiller; it was chopped and distributed to the feed bunks with the feed cart twice daily (at 09:00 h and 15:00 h). The particle size distribution of silage was like that of a typical total mixed ration (TMR).

Bulls and steers were fed the same diet and received 28 g dry matter (DM) of concentrate per kg of metabolic BW per day. The amount of concentrate offered to animals was adjusted at 14-d intervals based on their BW recorded every two weeks before the morning feeding. The animals were fed concentrate with different inclusion levels of protein (16% and 14% of crude protein in the concentrate for animals with BW of up to 300 kg and above 300 kg, respectively). The concentrate consisted of crimped triticale grain, rapeseed meal and mineral-vitamin premix for beef cattle (Cargill, Warsaw, Poland); up to 300 kg: 72.5%, 25%, 2.5%, and above 300 kg: 78.5%, 19%, 2.5%, respectively. The concentrate was offered in four identical portions per day, which could be consumed by animals at 4-hour intervals. Specific requirements were based on the INRA [14] guidelines for medium-early maturing young bulls with daily gains of around 1,000 g.

### Chemical analysis of diets

Silage samples (500 g) were collected from feed bunks twice a week and were stored at −20°C. The samples were pooled over 30-d periods and their nutrient content was determined according to the AOAC [15] procedure. The content of water-soluble carbohydrates was determined by the anthrone method [16]. The content of neutral detergent fiber, acid detergent fiber, and acid detergent lignin was determined by the method proposed by Van Soest et al [17] using the ANKOM^220^ fiber analyzer (ANKOM Technology Corp., Macedon, NY, USA), and protein nitrogen content was determined with the use of trichloroacetic acid [18]. Acid detergent insoluble nitrogen was determined based on the method proposed by Licitra et al [18], and buffer soluble nitrogen (BSN) – by the method described by Hedqvist and Udén [19]. The ammonium nitrogen content of silage was determined by micro-diffusion (modification of the method proposed by Conway [20], and acidity was measured with the HI 8314 pH meter (Hanna Instruments, Woonsocket, RI, USA). The concentrations of carboxylic acids in silages were determined by high performance liquid chromatography (HPLC) with the MetaCarb 67H P/N 5244 (Varian, Palo Alto, CA, USA) column and 0.0025 M sulfuric acid as the mobile phase, according to the manufacturer’s protocol. Biogenic amines in silages were identified by HPLC with a UV-VIS detector (546 nm) and the ET 125/4 Nucleosil 120–5 (Macherey-Nagel, Düren, Germany) C18 column, according to the method described by Joosten and Olieman [21]. The forage fill value of silages was calculated in the INRAtion 4.0 program [22]. The results of a chemical analysis of feed samples are shown in Table 1. They indicate that the quality of silage was typical of grass silage made without additives and used for feeding beef cattle in Poland [23].

### Behavioral data collection

Bunk visit data and silage intake for all experimental animals were recorded individually using the RIC system. Each animal had an ear tag containing a transponder, and each case of crossing the feed fence was recorded as a bunk visit. For each bunk visit, the system recorded the feed bunk number, the cow number, the initial and final times and weights of silage. Data were monitored continuously in each feed bunk. This electronic feeding behavior monitoring system had been validated for cattle fed TMR containing grass silage by Chapinal et al [24] who reported sensitivity and specificity of 100%. The recorded data were used to determine bunk visit frequency (frequency of feeding and non-feeding events), the duration of each visit and silage intake during each visit. The results provided a basis for calculating bunk attendance duration (total time of all daily feeding and non-feeding events, min/d) and feeding time (total time of all daily feeding events, min/d).

### Calculations and statistical analysis

The meal criterion, i.e. the minimum interval between feeding events defined as meals, was calculated. According to the literature [25], an individual meal criterion was calculated by fitting two exponential distributions (divided into a fast process and a slow process) to the frequency of log-transformed intervals between meals (Eq. 1):

$$Y_{t}=N_{f}\;\text{exp}\left(-\lambda_{f}t\right)+N_{s}\;\text{exp}(-\lambda_{s}t)$$

using the following equation (Eq. 2):

$$T_{c}=\left(\frac{1}{\lambda_{f}-\lambda_{s}}\right)\;\text{log}\;\left(\frac{N_{f}\lambda_{f}}{N_{s}\lambda_{s}}\right)$$

where: *Y_t_* is the frequency of intervals, *N_f_* and *N_s_* are the total number of intervals resulting from the fast process and the slow process, *λ_f_* and *λ_s_* are parameters of the fast process and the slow process. The intervals that were ≤3 s, i.e. three sampling periods in the data registration system, were rejected. The model parameters for calculating the meal criteria were determined using the optimization procedure *ga*, implemented in the MATLAB 2014a environment (MathWorks, Natick, MA, USA). The model coefficients were determined based on histograms of cumulative frequency (60 intervals) for three 20-day ranges (240±10; 360±10; 480±10), limiting the interval length to 200 min, because considerable changes in cumulative frequency were observed at an interval length of approximately 20 min. As a result, the use of an excessive number of points for estimating the second (slow) distribution could be avoided and both distributions could be satisfactorily fitted.

The parameters of exponential distribution, the coefficients of determination for the model described by Eq. 2 and the values of meal criteria (*Tc*) for three periods are shown in Table 2. The coefficients of determination for all three cases were approximately 0.99, pointing to good model fitting to the values determined based on cumulative frequency histograms.

Meal duration (min/meal) was calculated as the time between the beginning of the first feeding event and the end of the last feeding event, where intervals between the events were shorter than the meal criterion. Meal frequency (meals/d) was calculated by counting the number of meals per day. Meal size (g of silage DM/meal) was silage DM intake per meal.

An individual animal was a sampling unit. Measurement data were compared between age and gender groups—each animal was allocated to successive age groups and one of two gender groups (bull/steer). Silage dry matter intake (DMI), concentrate DMI, feeding rate of silage DM and average daily gain (ADG) were calculated for each animal. Mean BW during behavioral data collection was calculated based on the BW of animals recorded every two weeks.

A statistical analysis was performed using the Statistic Toolbox for MATLAB 2014a (MathWorks, USA). For the purpose of the statistical analysis, the animals were divided into three age categories: the youngest animals (7 to 10 mo of age), mid-aged animals (11 to 14 mo of age) and the oldest animals (15 to 18 mo of age). Breed was included as a random effect.

Since the data failed the assumptions of normal distribution and homogeneity of variance, the effects of age and gender (bull vs steer) on feeding behavior parameters in individual animals were determined by non-parametric Kruskal–Wallis (one-way analysis of variance on ranks) and Wilcoxon rank-sum tests (the number of groups higher than 2 and equal to 2, respectively). The groups were homogeneous but due to random factors (the removal of animals from the experiment), the number of measurements varied across groups. Therefore, tests with repeated measures could not be used. Differences were considered to be statistically significant at p value <0.05.

## RESULTS

The average BW of animals was 207±33 kg at the beginning of the experiment (180 to 220 days of age) and 474±71 kg at the end of the experiment (500 to 540 days of age). During the entire behavioral data collection period, mean BW was highest (p<0.01) in the oldest animals and lowest in the youngest animals (Table 3); mean BW was higher in bulls than in steers (p<0.01). Concentrate intake was lowest in the youngest animals and highest in the oldest animals, and it was higher in bulls than in steers (p<0.01). Due to random factors, the number of animals per pen ranged from 14 to 15 (14.83 on average per pen in each group) and the stocking density per feed bunk per group was 2.97.

### Effect of age on feeding behavior

Bunk visit frequency and meal frequency were affected (p< 0.01) by the age of animals (Table 3). The youngest animals were characterized by the highest frequency of RIC station attendance and meals, whereas the oldest animals were characterized by the lowest frequency of RIC station attendance and meals. Bunk visit frequency was 8.5% higher in mid-aged animals and 32.0% higher in the youngest animals compared with the oldest animals.

The age of animals significantly influenced bunk attendance duration, feeding time and meal duration. The average time spent by the youngest animals at the RIC station was 5.7 and 8.0 min longer, compared with the oldest and mid-aged animals. Feeding time was significantly shorter in the oldest animals than in the youngest individuals, but no significant differences in feeding time were noted between the youngest and mid-aged cattle. Meal duration was 9% shorter in the youngest animals than in the oldest animals.

Daily silage DMI, meal size and the feeding rate of silage were also affected (p<0.01) by the age of animals. Silage DMI per day and per meal was lowest in the youngest animals, which were also characterized by the lowest feeding rate of silage. Silage DMI per day and per meal was highest in the oldest animals, which were also characterized by the highest feeding rate of silage. Average meal size was larger in the oldest animals than in the youngest and mid-aged individuals (difference of 0.27 and 0.12 kg DM, respectively). The feeding rate of silage was higher in the oldest animals than in the youngest and mid-aged individuals (difference of 17.9 and 7.0 g DM/min, respectively).

### Effect of gender (bull vs steer) on feeding behavior

Bunk visit frequency and meal frequency were higher (p< 0.01) in steers than in bulls (difference of 31.3% and 8.85%, respectively) (Table 3). In comparison with bulls, steers spent significantly more time at the feeding station and devoted significantly more time (by 2%) to silage intake (p<0.01). Meal duration was also longer (p<0.01) in steers than in bulls (by 3.3%). Silage intake per meal was lower (p<0.01) in steers than in bulls (by 9.7%). Bulls were characterized by higher (p<0.01) daily silage DMI (by 3.4%) and silage feeding rate (by 4%) than steers.

## DISCUSSION

### Animal performance

In the present study, the mean BW of crossbred cattle increased progressively with age. However, the ADG achieved in this experiment was unsatisfactory, suggesting that their genetic potential had not been fully expressed when they were fed grass silage supplemented with small amounts of concentrate. Moreover, ADG values were only numerically higher in crossbred bulls than in steers, and they were lower than those reported by other researchers [26] who used feed rations with higher nutrient concentrations in purebred Charolais and Hereford bulls. In our study, the BSN content of grass silage was nearly two-fold lower than that noted in typical grass silages [26,27]. Furthermore, in our study the animals were housed in free-stalls, which probably led to greater energy losses in bulls due to mounting each other. Presumably, despite the higher intake of silage (by 150 g of DM/d) and concentrate (by 220 g of DM/d) by bulls, energy and protein intake was insufficient to fully express their growth potential.

### Feeding behavior

The bunk visit frequency determined in our experiment is consistent with that reported by Fitzsimons et al [12] in Simmental and Simmental×Holstein Friesian in-calf cows offered a grass silage diet. Average meal frequency in our study was also similar to that observed by other authors. Lancaster et al [10] studied the feeding behavior of Angus bulls and found that average meal frequency reached 7.7 meals/d at 6 to 8 animals per feed bunk. DeVries and von Keyserlingk [9] reported average meal frequency of 6.8 meals/d at 2 heifers per feed bunk.

The total daily feeding time reported in the literature varies considerably. In a study by DeVries and von Keyserlingk [9], average feeding time was 212 and 192 min/d in TMR-fed growing heifers under noncompetitive (1 heifer/feed bunk) and competitive (2 heifers/feed bunk) conditions, respectively, in groups of 8 animals. Similar results were reported by Fitzsimons et al [12] in pregnant beef cows at stocking density of approximately 2.8 cows per feed bunk and groups of 8 to 9 animals. In our study where stocking density was approximately 3 animals per feed bunk and the groups were nearly twice larger in size than in the cited studies, the total daily feeding time was twice shorter. Lancaster et al [10] demonstrated that in growing TMR-fed Angus bulls, average feeding time was approximately 100 min/d at feed bunk stocking density of 6 to 8 animals and very large groups (more than 50 animals), which indicates that feeding time may be correlated with group size.

According to DeVries and von Keyserlingk [9], competition at the feed bunk has a highly significant effect on the duration of a single meal, which lasts longer. However, in our study the average meal duration at stocking density greater than in the cited study was approximately only one-third as long as that reported by the above authors. Grant and Albright [28] suggested that in large groups, it is more difficult for animals to recognize group mates and their status in the social order. Therefore, aggressive behavior and displacements at the feed bank are encountered more frequently in larger, socially complex groups. In our experiment, a shorter total daily feeding time and shorter meal duration, compared with the findings of DeVries and von Keyserlingk [9], could result from a larger group size and displacements at the feed bunk. Another reason could be low palatability of grass silage, which was available in feed bunks, and the fact that the concentrate was offered separately. On the other hand, the small difference between bunk attendance duration and feeding time suggests that animals are not interested in spending time at the feed bunk when not eating.

The feeding rate of silage determined in our experiment is consistent with the feeding rates reported by DeVries and von Keyserlingk [9] in young Holstein heifers fed TMR with a high proportion of grass silage, and by Fitzsimons et al [12] in beef cows fed grass silage. In both cited studies, stocking density was somewhat lower than that in our experiment. In a study by Lancaster et al [10], the meal eating rate in bulls fed TMR containing maize silage, kept in large groups at stocking density of 6 to 8 animals per feed bunk, was twice higher than in our experiment. Similar observations were made by Proudfoot et al [29] who found that high competition for feed increased the rate at which dairy cows fed.

### Effect of age

An analysis of the effect of age on bunk visit frequency, meal frequency, duration and size revealed that younger animals more often visited the feed bunk, their feeding frequency was higher, but their meals were shorter and smaller in size. This is consistent with the findings of Forbes [30] who concluded that feeding frequency decreases while meal size increases as animals grow. The above could be due to differences in the BW and gut capacity of younger and older individuals. In cattle, feed intake is affected by the growth potential of animals, changes in their body composition at different stages of development [6], their age and BW as well as the age at which fattening begins [8]. Younger animals feed more frequently than older individuals probably because they have a less developed gastrointestinal tract and consume smaller meals. The higher bunk visit frequency in younger animals may also be explained by the lack of a stable hierarchy in a newly-created group, which could contribute to more frequent displacements at the feed bunk [28]. According to Zobel et al [31], bunk visit frequency is highly significantly correlated with aggressive behaviors. In our experiment, the animals were systematically observed but competitive behaviors were noted only for 15 to 20 minutes after delivery of silage. Our results are consistent with the findings of Greter et al [32] who found that competition for feed is particularly noticeable when fresh feed is delivered. Thus, the provision of fewer feeding places than animals may lead to competition for feed, particularly during peak periods of feeding activity [33,34]. The higher bunk visit frequency noted in the youngest animals could also result from their greater curiosity. Young individuals are also more susceptible to social facilitation. The highest difference between bunk attendance duration and feeding time in younger individuals could also be related to greater curiosity in this group of animals. In our study, highly significant differences were noted in the majority of feeding behavior traits between animals of different ages, which suggests that age is an important determinant of feeding patterns in cattle.

### Effect of gender (bull vs steer)

Our findings show that gender (bull vs steer) affects feeding behavior in cattle fed grass silage. Significantly higher bunk visit frequency, meal frequency and longer meal duration in steers in the present study indicate that the feeding behavior of cattle is affected by sex category. Steers spent more time at the feed bunk and devoted more time to silage intake, but their meals were smaller in size and the feeding rate of silage was lower. Bulls consumed silage less frequently and for a shorter time, but at a faster rate. Therefore, they consumed larger amounts of silage per meal and per day. The differences in feeding behavior between bulls and steers could be due to higher levels of testosterone and appetite-related hormones in the former [35]. Another reason could be greater feed bunk competition observed in bulls, also resulting from their higher testosterone levels. It also appears that bulls spent less time at the feed bunk due to their non-feeding activities including physical activities [7] and more frequent sexual activities [36]. Sexual and physical activities increase energy expenditure and could stimulate appetite. Our results corroborate the findings of Devant et al [7] who analyzed young (5 to 10 mo of age) Holstein bulls and steers fed straw and concentrate diets, thus suggesting that gender affects feeding behavior regardless of feeding regime.

## CONCLUSION

The results of this study indicate that age and gender (bull vs steer) exerted significant effects on the feeding behavior of young beef cattle. Bunk visit frequency and meal frequency decreased, whereas the feeding rate of silage, and the average duration and size of a single meal increased with age. Bunk visit frequency and meal frequency were significantly higher in steers than in bulls. Daily feeding time was longer in steers than in bulls, but the feeding rate of silage was lower in steers, and their meals were longer in duration but smaller in size. Due to a slower rate of roughage intake, steers should be allowed to spend more time at the feed bunk than bulls. Further studies investigating feeding behavior in cattle are needed to determine the optimal number of animals per roughage feeding station, depending on their age and gender.

## Figures and Tables

**Table 1:** Characteristics of silage offered to cattle in the study (mean±SE)

Item	Value
No. of samples	26
DM1) (g/kg)	376±2.4
pH	4.65±0.01
Composition of DM (g/kg)	
OM	921±0.4
CP	151±0.5
NDF	566±1.8
ADF	357±1.6
ADL	48.4±0.38
WSC	74.6±1.46
Nitrogen compounds (g/kg total N)	
Protein nitrogen	570±2.8
BSN	279±2.4
ADIN	96.1±1.0
Ammonium N	60.9±1.5
Carboxylic acids (g/kg DM)	
Lactic acid	43.2±0.76
Butyric acid	5.17±0.1
Biogenic amines (mg/kg DM)	
Tyramine	194±4.3
Putrescine	430±10.8
Cadaverine	250±7.6
Estimated NE (UFV2)/kg DM)	0.756

SE, standard error; DM, dry matter; OM, organic matter; CP, crude protein; NDF, neutral detergent fiber; ADF, acid detergent fiber; ADL, acid detergent lignin; WSC, water-soluble carbohydrates; BSN, buffer-soluble nitrogen; ADIN, acid detergent insoluble nitrogen; NE, net energy.

1) Corrected for loss of volatiles during oven-drying.

2) Feed units for meat production (INRA) [14,22].

**Table 2:** Parameters of exponential distribution, the coefficients of determination and the values of meal criteria in three periods

Point/period (d)	Model coefficients (distribution parameters)				R^2^	Tc5) (min)

	N_f_1)	λ_f_2)	N_s_3)	λ_s_4)		
240±10	2.5320	0.0027	2.1781	0.1412	0.9895	11.9
360±10	3.0163	0.0021	2.3006	0.1499	0.9907	12.0
480±10	2.9007	0.0022	2.2546	0.1487	0.9906	11.7

R^2^, coefficient of determination.

1) Total number of intervals for the fast process.

2) Parameters of the fast process.

3) Total number of intervals for the slow process.

4) Parameters of the slow process.

5) Values of meal criteria.

**Table 3:** The effect of age and gender (bull vs steer) on the performance and feeding behavior of young beef cattle (mean±SD)

Item	Age (months)			Gender (bull vs steer)	
	
	7–10	11–14	15–18	Bulls	Steers
Number of animals	180	178	176	90	90
ADG (g/d)	905±282	924±254	951±300	972±118	933±145
Mean BW (kg)	259^C^±44	347^B^±54	451^A^±74	390^A^±83	381^B^±101
Bunk visit frequency (visits/d)	53.3^A^±28.3	43.8^B^±21.7	40.3^C^±20.7	37.4^B^±18.8	49.1^A^±24.2
Meal frequency (meals/d)	9.20^A^±3.48	8.23^B^±3.05	8.10^C^±2.80	7.99^B^±2.93	8.63^A^±3.11
Bunk attendance duration (min/d)	111.4^A^^a^±55.2	103.4^B^±53.2	105.7^A^^b^±42.7	103.1^B^±49.7	107.6^A^±47.8
Feeding time (min/d)	102.8^A^±51.4	99.5^A^±51.3	102.7^B^±41.6	100.3^B^±48.3	102.3^A^±45.6
Meal duration (min/meal)	17.3^C^±16.4	18.3^B^±16.6	19.0^A^±16.7	18.1^B^±16.0	18.7^A^±17.1
Feeding rate of silage (g DM/min)	36.9^C^±16.4	47.6^B^±27.3	54.8^A^±24.5	50.8^A^±21.2	48.8^B^±29.0
Meal size (g of silage DM/meal)	365^C^±321	513^B^±451	634^A^±536	566^A^±501	511^B^±457
DMI of silage (kg DM/d)	3.38^C^±1.86	4.18^B^±2.33	5.15^A^±2.30	4.62^A^±2.60	4.47^B^±2.25
DMI of concentrate (kg DM/d)	1.85^C^±0.54	2.32^B^±0.70	2.64^A^±0.82	2.52^A^±0.80	2.30^B^±0.71

SD, standard deviation; ADG, average daily gain; BW, body weight; DM, dry matter; DMI, dry matter intake.

A–C Mean values within a row marked with different uppercase superscript letters differ significantly at p<0.01.

a–c Mean values within a row marked with different lowercase superscript letters differ significantly at p<0.05.
